# Effects of Three Music Therapy Interventions on the Verbal Expressions of Children With Autism Spectrum Disorder: A Combined Single-Subject Design

**DOI:** 10.3389/fpsyg.2022.819473

**Published:** 2022-03-04

**Authors:** Nayla Attar, Anies Al-Hroub, Farah El Zein

**Affiliations:** ^1^Department of Education, American University of Beirut, Beirut, Lebanon; ^2^Emirates College for Advanced Education, Abu Dhabi, United Arab Emirates

**Keywords:** ABA, Music Therapy, ASD, combined single-subject research design, multielement design, verbal expression, non-verbal

## Abstract

The specific aims of this research study were to (a) examine the differential effect of three different music interventions, namely the interactive music playing therapy (“music and singing”), interaction music singing therapy (“singing”), and receptive music therapy (“listening”) studying the varying latency periods in the response time it took 3-year-old children diagnosed with autism spectrum disorder (ASD) to elicit the target word vocally; and (b) assess the index of happiness of children with ASD after the implementation of the three music interventions, which can, in turn, be used to influence their overall quality of life through this specific intervention. This study used a combined single-subject research design consisting of delayed multiple baseline across the participants and a multielement design to compare the effects of each music intervention technique targeting the child’s verbal response during playback of a practiced song. Findings demonstrated “singing” to be associated with the lowest latency compared to the other two interventions (“listening” and “singing and music”) across participants. Additionally, happiness levels varied from neutral to happy, signifying an overall positive experience during participation in the music applied behavior analysis (ABA) intervention.

## Introduction

Communication and social skill deficits are two critical impairments characterizing children with autism spectrum disorder (ASD) along with restricted and repetitive behaviors, interests, or activities (American Psychiatric Association [APA], 2013). ASD usually manifests in early childhood and persists through life (American Psychiatric Association [APA], 2013). Interventions based on the science of applied behavior analysis (ABA) are supported by extensive empirical research (Reschke-Hernández, 2011) developed to teach commonly deficient skills such as complex communication, social, play, and self-help skills (Leaf and McEachin, 1999). Because children with ASD have difficulties completing everyday tasks, ABA breaks down skills into manageable tasks then builds on them so that a child can learn in the natural environment. When ABA is delivered in a fun, playful, and positive way, it can be an ideal means to help a child learn functional skills (Hall and Isaacs, 2011). Since ABA aims to analyze and modify a child’s behavior, music can provide stimulation for the positive reinforcement of the desired behavior (Liao, 2013). Given the therapeutic impact it has on individuals with communication and social skill deficits, music is an ideal intervention to use for children with ASD (Mendelson et al., 2016).

Despite the abundance of research on ASD worldwide, the Eastern Mediterranean Region is lagging in research on ASD in general (Chaaya et al., 2016; Luor et al., 2021). Research published in JABA between the years 2007 and 2012, out of 112 studies on ASD, only one was conducted in a developing country (Ulke-Kurkcuoglu and Kircaali-Iftar, 2010). The findings from these reviews indicate that ABA, music therapy, and other intervention programs tailored to ASD need to be implemented in developing countries, including Lebanon, where ASD is reported as high prevalence among the few small-scale studies conducted (Chaaya et al., 2016). Other noted helpful and accessible interventions for ASD of which include music therapy, are also rare to find in Lebanon. The most considerable evidence that stands for the lack of ABA application is the little research that has been published regarding the use of ABA in Lebanon (Daou, 2014), and on the use of music therapy.

Because communication is difficult for children with ASD, using an intervention that teaches responding with an immediate and high reinforcement even at attempts at speech may demonstrate the connection between responding and reinforcement, reducing avoidance behavior (Koegel et al., 2001).

In the past five decades of its documentation through single-subject methodology, ABA procedures repetitively showed improvement in the behaviors of children with ASD (Sambandam et al., 2014). Motivational improvements incorporated into intervention programs like language or teaching programs have been evidenced to increase socialization, such as verbal interaction for children with ASD exhibiting social interaction deficits (Grove et al., 2015). Results of multiple studies show that when children were consistently reinforced for their attempts at a speech rather than for accuracy of speech production, they made more rapid and consistent progress, which were maintained over time (Koegel et al., 2001).

The goal is to make motivation something that the child self-initiates so that he is always learning in the environments around him (Koegel et al., 2001). Therefore, a vital intervention goal should concentrate on motivation, which will, in turn, be more likely to receive a response from the child so that the child later self-initiates responses to receive self-motivation from everyday learning experiences from social, linguistic, and academic interactions. This can increase the number of stimuli input for potential learning opportunities to the child throughout the day.

### Music Therapy and Autism Spectrum Disorder

Internationally, using music therapy with children who have ASD is a major area for practice in music therapy (Dimitriadis and Smeijsters, 2010; Rickson et al., 2015). In a survey of 328 professional members of the American Music Therapy Association about 27.3% of respondents reported that of all their clients, children with ASD made up between a quarter and one-half (Kern et al., 2013).

There have been varieties of approaches in music therapy, which have developed over time for children with ASD (Reschke-Hernández, 2011). Multiple reviews about music therapy practice with ASD have been published including meta-analysis studies (e.g., Geretsegger et al., 2014; Li et al., 2016). Results from the meta-analysis studies conducted by Geretsegger et al. (2014) and Li et al. (2016) showed that music therapy is an effective intervention in improving social skills, and verbal communication for children with ASD. Previous studies also suggest that music therapy protocols that involve singing and listening to songs may increase appropriate behaviors, social communication skills, and reduce stereotypy in individuals with ASD (Gee et al., 2014; Paul et al., 2015; Dieringer et al., 2017). On the other hand, in a more recent study conducted by Bieleninik et al. (2017) with large sample size and a group experimental research design, music therapy did not demonstrate benefit to participants with ASD when compared to a control group. Similarly, several single-subject research design studies were not able to show significant improvements in social and language skills in individuals with ASD upon implementation of music therapies (Corbett et al., 2008; Lim, 2010; Schwartzberg and Silverman, 2013). These inconsistent findings regarding the effects of music therapy on individuals with ASD highlight the need for further assessment of this type of treatment.

Little research was done on ABA and Music therapy techniques being used together with ASD. Lim and Draper (2011) study, for example, explored how the function of language itself provides positive reinforcement for ASD and referred to this process in the study as ABA Verbal Behavior (ABA VB). Language can be sorted into four different categories based on function including mand (requesting), tact (labeling), echoic (imitating), and intraverbal (used in social interaction or conversation). The ABA VB approach explored how each verbal operant is associated with independent functional control. This study used various musical instruments as effective antecedent variables for reinforcement in ABA VB language training. The findings revealed that singing did serve as an effective antecedent variable and automatic reinforcer with ABA VB training in verbal enhancement. Although Lim and Draper (2011) study does tie together music therapy, ABA VB, and communication, it does not show how music therapy and ABA can decrease the response time a child may take to speak after the given musical reinforcement.

The music therapy interventions used in the current study, therefore, were intended to enhance verbal expression in children with ASD. These interventions are unique techniques designed to motivate the child with ASD using musical reinforcement while simultaneously targeting speech attempts at target words throughout unfamiliar songs. Using a combination of behavioral methods, motivation, and music, all three have proven highly rewarding and successful for children with ASD by research setting the stage for potentially productive and fun learning sessions.

Most children with ASD do seem to have a strong preference for music according to research (Li et al., 2016); however, to uncover which is most enjoyable for each child, one would have to observe affective behavior (Parsons et al., 2012). A benefit of using the index of happiness measure during activities for children with ASD is that stakeholders (e.g., researchers, practitioners, and parents) can use the best techniques to increase the happiness level of students. This technique can, in turn, be used to influence their overall quality of life (Bobzien, 2014). The quality of life or happiness of individuals with disabilities has not been as much explored as the other variables, like, for example, skill acquisition (Davis et al., 2004).

An intervention program can be proven effective, and may even supply a high index of happiness measures, but that does not necessarily mean it is appropriate for implementation (Carter and Wheeler, 2019). Social validity data were collected at the end of the implementation of the three music interventions. The inclusion of social validation can provide important evidence to support the use of specific evidence-based practices (EBPs) for children with ASD across the life span. Therefore, more of it is needed in research. Because of its importance toward proper EBP implementation, direct inclusion of social validity was reviewed in research articles and was only found in 27%. The findings demonstrate that the overall evidence for social validity is limited.

The specific aims of this study, therefore, were threefold: (a) examine the differential effect of three different music interventions, namely the Interaction music singing, interactive music playing, and Receptive Music Therapy (RMT). The researchers examined the varying latency periods in the response time it takes three children with ASD to vocally elicit the target word; and (b) assess the index of happiness of children with ASD after the implementation of the three music interventions, which can, in turn, be used to influence their overall quality of life through this specific intervention.

## Research Design

This study used a combined single-subject research design consisting of delayed multiple baseline across participants and multielement designs. The multiple baseline design allowed for the demonstration of experimental control as the intervention was introduced to each participant at a different point in time (Kennedy, 2005). Additionally, the multielement design allowed for a comparison of the effects of each music intervention technique.

### Research Questions

The primary research questions that guided this study were: (a) How do the three music interventions compare in their impact on decreasing the latency period preceding a vocal response in three children with ASD?, and (b) Do children with ASD show different levels of happiness from participation in the three music interventions?

### Participants

#### Sampling Procedure

This study used a purposive sample, including children diagnosed with ASD from the Lebanese Autism Society (LAS) aged 6–7 years old. Participants were selected from a group of children diagnosed with ASD who attended an inclusive private school as part of the LAS inclusion program. Given the nature of social communication skills that are targeted in this study, children in this age range were preferred. In addition, this target age shares similar daily experiences in the school setting for a broader generalization of the results. Verbal and non-verbal children were included. The criteria for selection included the following: (a) *Diagnosis of ASD used in DSM-5*: The participants had a formal diagnosis of the ASD specified by a certified pediatrician, psychiatrist or psychologist, and (b) *Understanding of the English language*: The participants were able to understand the English language, the language of communication, and were chosen from those enrolled in the English section of LAS.

Potential participants were excluded if they met one or more of the following criteria:

(a) *Sensory disability:* Children with a co-occurring sensory disorder such as blindness or deafness were excluded because this would change the implementation procedures of the study.
(b) *Previous experience of music therapy:* To link effects from the study strictly to the implementation of the study, participants included did not have previous experience in music therapy.
(c) *Intellectual disability:* Children with co-occurring intellectual disabilities were excluded from the study.
(d) *Speech disorder:* Children with speech disorders were excluded.

The above-mentioned exclusion criteria were identified to obtain a group of participants with similar ASD-related characteristics. Following the above criteria, questions regarding first language, the primary language used at home, speech problems, speech therapy, hearing problems, visual problems, and intellectual ability were addressed, in the form of a questionnaire, so that the conditions for all children are more so identical before initiating the study. The school administrator selected five participants after they were given the set of criteria set by the researchers. From the five, the researchers selected the three most identical cases to generalize results better.

#### Description of Participants

##### Youssef

Youssef was a 6-year old verbal male in KG3 (a preschool for children age 5 to 6) with a diagnosis of ASD from a neurologist. Youssef had speech problems and benefited from speech therapy. He had a shadow teacher that stays beside him during the day. The main languages spoken at home were reported to be Arabic and English while their primary language is English. He had no prior experience with music therapy.

##### Andy

Andy was a 6-year old non-verbal male in KG3 (a preschool for children age 5 to 6) with a diagnosis of ASD from a neurologist. Andy attends a multi-disciplinary approach, including speech, ABA, and psychomotor sessions. He had a shadow teacher at school that always stayed by his side. The main languages spoken at home were reported to be Arabic and English, and Andy’s primary language is English. He had no prior experience with music therapy.

##### Alex

Alex was a 6-year old non-verbal male in KG3 (a preschool for children age 5 to 6) with a diagnosis of ASD from a neurologist. Alex used to be verbal and later became non-verbal. He received a diagnosis of ASD from a neurologist. Andy attends a multi-disciplinary approach, including speech, ABA, and psychomotor sessions. The main languages spoken at home were reported to be Arabic and English, and Andy’s primary language is English. He had no prior experience with music therapy. Approval was granted from the Institutional Review Board before the study commenced. Approval of the study was also gained from the school principal for the research site.

### Dependent Variables and Data Collection

Three dependent variables were measured to answer the research questions addressing verbal expression, and index of happiness. Additionally, interobserver agreement (IOA) data were collected for verbal expression and index of happiness measures.

#### Verbal Expression

The verbal expression refers to the participant’s response to a given song after the song is paused. The operational definition of verbal expression varied between verbal and non-verbal children:

##### Verbal Expression for Non-verbal Children

This behavior is measured when the child opens their mouth to produce a vocalization, approximately mimicking the target word. Mimicking the target word includes either pronouncing the same number of syllables and/or producing similar vowel tones to that of the target word (Example, if the target word is “star” and the child expresses “ta,” it is one syllable and also embodies similar vowel tones to the original target word, “star”). Verbal expression is counted following a pause directly after a musical stimulus. Therefore, in ABA terminology, the antecedent is the different musical stimulus being played, the target behavior is the verbal expression as defined by the child, and the consequence is the continuation of music by the type of behavior displayed. The time between the antecedent variable and the emitted target word/sound will vary and is significant to this study.

##### Verbal Expression for Verbal Children

This behavior is measured when the child correctly articulates the target word following a pause directly after a musical stimulus. Therefore, in ABA terminology, the antecedent is the different musical stimulus being played, the target behavior is the verbal expression as defined by the child, and the consequence is the continuation of music by the type of behavior displayed. The time between the antecedent variable and the emitted target word/sound will vary and is significant to this study.

A response latency recording system was employed to measure verbal expression. Response latency has been used in previous studies to measure the level of motivation (Koegel et al., 2010) and affective state (Chaspari et al., 2013) during task engagement of children with ASD. The chosen target behavior for this study was the spoken word/sound following the antecedent variable, as previously defined. The antecedent variable differed depending on the type of independent variable (music technique) being used. The time between the antecedent variable or the stimulus and the emitted target vocal response from the student is significant. For that reason, the most suitable method of data collection for verbal expression was latency recording. Latency was measured utilizing the timer on the video recording starting directly after the stimulus and stopping either when the target behavior was displayed or after a maximum of 6 s. In the case when the child failed to pronounce the target word, the researcher gave a 6-s delay prompt (Leaf and McEachin, 1999) and then proceeded to trial the next target word in the song. An incorrect response was also recorded as 6-s, as well as a late response. The observer then timed the new trial to measure the latency period for the next target word. The observed data for each video-recorded session on the latency period was then collected using pen and paper. The latency periods for each individual were collected and compared. Latency recording during each music practice helped determine the preferable practice for promoting verbal expression for each child individually, answering the first experimental question.

#### Index of Happiness

In an attempt to uncover the effect of the three music interventions on the activity enjoyment and to learn which of these interventions is most enjoyable for each participant, we observed their affective behavior (Parsons et al., 2012). Index of happiness measurement was adopted from Dunlap and Koegel (1980) scale. The scale defines happy as “smiles, laughs appropriately, seems to be enjoying self,” neutral as “may smile or frown occasionally but does not appear to be decidedly happy or particularly unhappy,” and “unhappy as cries, pouts, tantrums, appears to be sad, angry, or frustrated child seems not to be enjoying self” (p. 622).

Rating Scales for Child Affect (Interest and Happiness); (Dunlap and Koegel, 1980) was used to assess interest and happiness during the study phases. The tool has been adapted in current studies by Koegel et al. (2013). It has also been referred to in various recent works such as Dunlap et al. (2010) and Lang et al. (2016).

The mentioned rating scale is a six-point Likert scale composed of four areas, including enthusiasm, happiness, interest, and general behavior (Dunlap and Koegel, 1980). For this study, only the scale for happiness is relevant. The categories of the scale are divided into two points each. Depending on the adapted scale’s definitions of happiness, the observers rated either a 4 or 5 according to the observed intensity. Marking a neutral rating would be recognized as the occasional smile or frown in which the observers rated either a 2 or 3 depending on the extent of happiness. Rating unhappiness would be either a 0 or 1 depending on if the intensity of the child’s behavior reflected as crying, pouting, or screaming. A 2-min recording segment was withdrawn from previously recorded material of the sessions and rated. From the 2-min recording segment, 40-s intervals from each intervention were scored. The researchers used the partial-interval recording system to score the occurrence of happiness-related behavior of participants during the use of each intervention for every 2 min. If the display of happiness or unhappiness occurred throughout any period of the interval, the interval was scored as an occurrence.

#### Interobserver Agreement (IOA)

To account for IOA, two observers independently recorded verbal expression and index of happiness data using 30% of the total video recordings for each participant’s baseline and intervention phases. The observers were a university professor who specializes in ABA and a graduate student who received extensive training in latency recording for verbal expression and partial interval recording for the index of happiness. Interobserver agreement data was calculated by dividing the number of agreements by the number of agreements plus disagreements and then multiplying the resulting number by 100 to convert the result to a percentage (Kennedy, 2005). Overall, latency recording IOA scores were 90% for Youssef, 94% for Andy, and 98% for Alex. Index of happiness agreement scores were 100% for Youssef, 92% for Andy, and 96% for Alex.

#### Social Validity

Following the termination of intervention with the three participants, social validity was measured. Social validity is defined as “consumer satisfaction with goals, procedures, and outcomes of programs and interventions” (Callahan et al., 2017, p. 189), with consumers being the parents and teachers of the participants undergoing the intervention as they are too young to answer themselves. It is critical to note that an effective procedure that is not socially acceptable is more likely to be replaced by educators and service providers with a less effective but more socially acceptable alternative (Langthorne and McGill, 2011). Researcher-developed questionnaires and scales have been widely utilized in intervention studies to assess the social validation of the intervention being evaluated (Lancioni et al., 2006). The researchers of this study developed a social validity rating scale to assess the parents’ and teachers’ perspectives in regards to meeting the goals, procedures, and outcomes of the study interventions. The Likert scale contained twelve questions that ranged from “not at acceptable” to “very acceptable.”

### Study Phases

Sessions took place individually on a one-on-one basis with the first researcher, who is also an ABA therapist. Since the researchers in this study used music as an interventional method to enhance verbal expression, to measure the index of happiness, and to measure social validity, the session length was 20 min long. It took place two times a week for 2 months. The researchers aimed to come 15 times but reached a total of 14 times over 8 weeks, approximately 38 days. The sessions took place during the same time of day for each individual to control for the environmental conditions of the experiment (Byiers et al., 2012). Each music intervention during the 20-min session was conducted for a period of 6-min each. The order of the interventions was structurally rotated to control for preference. Table 1 below illustrates the structural order of intervention as an example over 3 days. The intervention was rotated over 39 days, which is a multiple of 3 so that the allotted time can be rotated fairly.

**TABLE 1:** Example of intervention order given over 3 days.

Day 1	Day 2	Day 3
Singing 10 min	Listening 10 min	Music and singing 10 min
Music and singing 10 min	Singing 10 min	Listening 10 min
Listening 10 min	Music and singing 10 min	Singing 10 min

Employing a combined single-subject design allowed a display of multiple baselines across participants and a multielement design (Kennedy, 2005). The effects on response latency from the interventions were analyzed by comparing the data obtained during baseline with the data collected from the music intervention sessions allowing analysis of the interventions on response latency for each participant. The use of the multielement design allowed a comparison to be made between the effects of each music intervention on response latency.

#### Pre-baseline

A phase known as the pre-baseline took place before the actual baseline. Through the intervention, participants learned unfamiliar songs; therefore, a phase was necessary to teach, familiarize, and acclimatize them with the target words and melodies before baseline measures were taken. The researchers conducted this phase by playing the songs in their entirety without any pauses in the music so that the child participants become familiar with all the words, target words included. In this way, they were familiarized with the words used in the baseline phase and later in the intervention. It was kept in mind that each child differed when it came to the ability to carry a tune or to learn words (Honig, 1995).

#### Baseline

The baseline for the latency period was measured before the formal implementation of the study to compare the effects of the intervention. The researchers measured the baseline by pausing the music before the target words in the song played on the laptop. The latency period was timed with a stopwatch after the musical antecedent stimuli were played to the children and were stopped after giving the child a maximum latency for a response of 6 s, with 6 s corresponding to no response. Any correct response of the target word elicited before 6 s was recorded. Incorrect answers were also recorded at the 6-s mark as no response.

#### Music Therapy Interventions

Since a musical intervention is permitted to incorporate music therapy principles (Kern et al., 2013), some aspects from classical music therapies were incorporated into the intervention designs for the study. They consist of both active and passive methods (Prakash, 2015), including interactive singing, interactive music playing (Kaplan and Steele, 2005), and receptive music therapy (Accordino et al., 2007).

##### Interactive Singing (Signing Intervention)

It was the first music intervention that was implemented in this study. Throughout this study, it will be referred to only as “singing.” Familiar and non-familiar songs will be set within a predictable framework. The songs were made to be repetitive and used clear language. The tempo, volume, pausing, and wording are interchangeable during the song in response to the child. Dramatic pauses during songs offer opportunities for the child to respond during an interactive musical melody. Soon the child may anticipate the pauses in the song as a pattern posing a higher likeliness for him or her to respond. In this way, the child and first researcher mimicked an everyday state-response conversation.

##### Interactive Music Playing (Music and Signing Intervention)

It involved the first researcher and child playing different selected musical instruments such as the keyboard, the drum, the maracas, or rhythm sticks while singing familiar and non-familiar songs set in a predictable framework. Pauses throughout the songs accompanied by the instruments were made for the child to anticipate pauses and respond during the interactive melody. Previously composed and structured unfamiliar songs were used for this musical intervention. It will be referred to as “music and singing” throughout this study.

##### Receptive Music Therapy [RMT] (Also Known as Listening Intervention)

It involved listening to live or recorded music (Accordino et al., 2007). Previously recorded unfamiliar music set with words were played on the radio to enable opportunities for the child to listen and respond following a pause in the music. It will be referred to as “listening” throughout this study.

### Session Structure

**FIGURE 1:**
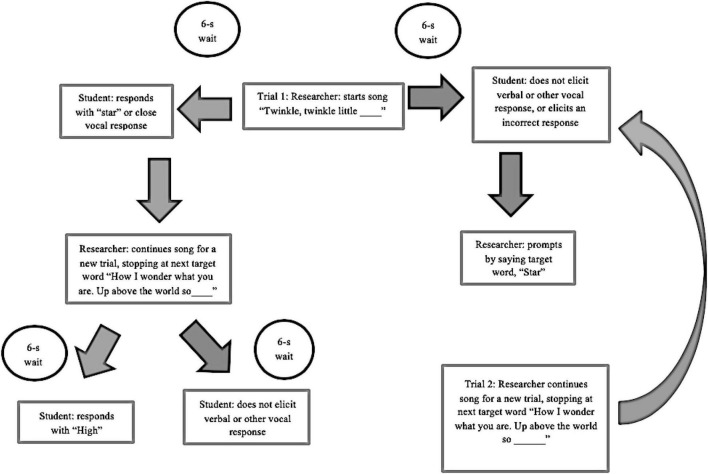
Diagram for the ABA/music intervention.

The goal of the intervention was for the child to elicit the target behavior, a vocal response following the presentation of an antecedent variable stimulus signified by the first researcher purposely pausing one of three ongoing musical techniques. The first researcher, who is a practicing ABA therapist, conducted the joint ABA and music sessions. The researcher had beforehand highlighted the stimuli or the words/phrases that were used to target verbal responses from the children during the interventions. Six minutes were dedicated to each musical technique for a total session time of 20 min. Each time the researcher began conducting “singing,” “singing and music,” or “listening” and paused the intervention, the stimulus signaling the child to elicit the target word, was considered a trial. There were two trials per song or two missing target words per song. There were six songs total, with each song being approximately 2 min in length. Each day the intervention was run, the same songs were used to allow equal opportunities for target behaviors to be exhibited across the three different music techniques. Song choice included six unfamiliar songs in order to omit possibly biased responses from previously learned songs (Figure 1).

#### Applied Behavior Analysis Trial Composition

The structure of this intervention followed ABA techniques assuming music as the reinforcement. Trial 1: The first researcher began playing the first song. Upon approaching the target word, the researcher sang the stimulus and paused the music allowing time for the child to fill in the target word. Usually, a 2–3 s delay prompt is given for a child to respond in ABA (Leaf and McEachin, 1999). However, according to one certified music therapist, plenty of time should be given during music therapy for a child to process the information and respond (Judd, 2015). Therefore a 6-s delay prompt was given. If the child was successful in the trial for eliciting a verbal response, the first researcher provided verbal praise, resumed the music, and commenced a brand new trial for the next target word. However, if the child was not successful after 6 s or did not respond, the researcher gave a prompt (Leaf and McEachin, 1999), which was singing the target word aloud for the child to hear. The same musical strategy was then repeated for the second target word. Incorrect answers were recorded at the 6-s mark as no response.

## Data Analysis

**FIGURE 2:**
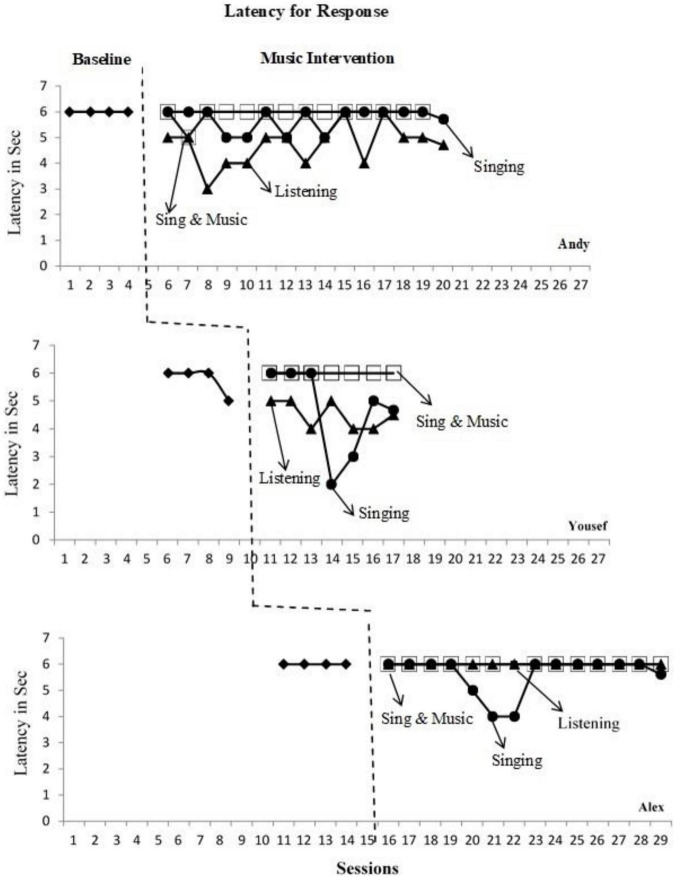
Latency of response scores reported in number of seconds.

Sessions are represented on the *x*-axis, and the *y*-axis represents the latency period, or the response time preceding verbal expression (see Figure 2). Researchers have conventionally used the visual analysis method to interpret single-subject research design studies (Horner et al., 2005). Figure 2 allowed for a visual inspection of performance on the occurrences of verbal expression to observe a differential experimental effect on the students’ response time across three musical interventions each. Each dependent variable was analyzed based on visual inspection of the graphs using (a) level, (b) variability, (c) trend, and (d) overlap (Kratochwill et al., 2013) to estimate the effect of the changes between baseline and intervention. Each child’s mean scores or levels within the two phases (baseline and intervention) were used to compare the effect of each intervention on the quickness of the response time. The line of best fit for the intervention data was used to describe whether the data had an ascending, descending, or neutral pattern.

Lastly, the overlap was calculated using a procedure called “percentage of non-overlapping data” (PND) for quantitative analysis of the data (Scruggs and Mastropieri, 1998). The PND was found by identifying the number of intervention data points that are lower than the lowest data point in the baseline, divided by the total number of intervention data points, times 100. According to Scruggs and Mastropieri (1998), a highly efficient intervention has a PND score equal to or larger than 90% when it is equal to or larger than 70% but smaller than 90% it is an effective intervention, between 50 to 70% is a questionable intervention, and below 50% is an ineffective intervention (Heyvaert et al., 2015). This technique was chosen because it illustrated the consistency of each child’s response time with each of the interventions as compared to their original response times at the baseline level.

## Results

Results from the music intervention indicated that Youssef achieved a decrease in the latency period, with number 1 being the intervention yielding the quickest response: (1) “singing,” (2) “listening” (3) “singing and music.” Andy’s latency in responding decreased the quickest during (1) “listening,” (2) “singing,” (3) “singing and music.” As for Alex’s latency in responding, it was shown to decrease the quickest during “singing” while “singing and music” and “listening” equally showed no effect in reducing his response time.

### Youssef

**FIGURE 3:**
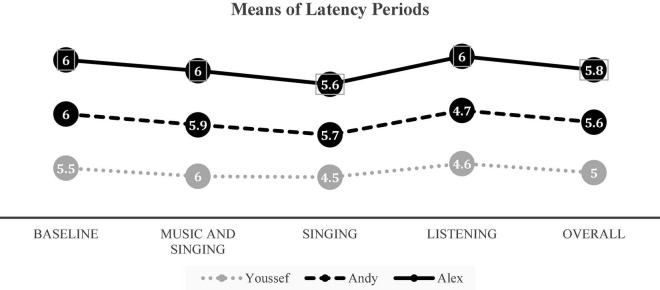
Means of latency for the three participants during each of the musical interventions.

Youssef’s latency of responding to target words had a mean of 5.5 s during baseline, as displayed in Figure 3. His mean score decreased to 5 s during the musical intervention phase. Visual analysis of his latency of responding graph showed a decrease in his response time upon implementation of both the “singing” intervention and the “listening” intervention (see Figure 2). Youssef’s mean scores were 4.6 s during the “listening” intervention and 4.5 s during the “singing” intervention. However, during the “singing and music” intervention, Youssef’s latency of responding remained at 6 s, where 6 s corresponds to “no response.” During baseline, Youssef’s scores on the latency of responding ranged from 6 to 5 s, with 6 s being the most frequent latency of responding. His responding time during the “singing” intervention varied from 6 to 2 s, with 6 s being the most frequent latency of response time. His responding time during the “listening” intervention varied from 5 to 4 s, with the frequency of latency response being equal for both 5 and 4 s. During the “listening” intervention, his response time did not vary but stayed constant at 6 s throughout. Additionally, visual inspection of Youssef’s latency for response graph reveals clear descending trends during the “singing” and “listening” interventions demonstrating a quicker response rate to the target words during these musical interventions. However, a neutral trend is displayed during the “singing and music” intervention.

Youssef completed the intervention by learning all the target words to the songs and was observed to be restless by the end of the intervention. He exhibited some challenging behaviors, such as trying to exit the room. The researchers felt the need to end the intervention early since all the target words had been mastered with quick response time.

### Andy

Andy’s latency of responding to target words had a mean of 6 s during baseline, as displayed in Figure 3. His mean score decreased to a mean of 5.6 s during the three musical intervention conditions collectively. Visual analysis of his latency for response graph showed a clear decrease in his response time upon implementation of the “listening” intervention (see Figure 2). Andy’s mean scores were 5.7 s during the “singing” intervention and 4.7 s during the “listening” intervention. During the “singing and music” intervention, Andy’s latency of responding was 5.9 s. During baseline, Andy’s latency for response stayed constant at 6 s, with 6 s corresponding to “no response.” His responding time during the “music and singing” intervention varied from 6 to 5 s, with 6 s being the most frequent latency for response time. His responding time during the “listening” intervention varied from 3 to 6 s, with 5 s being the most frequent latency for a response. During the “singing” intervention, Andy’s response time similarly varied from 6 to 5 s, with 6 s being the most frequent latency for response time. Visual inspection of Andy’s latency for response graph reveals increasing trends for “music and singing,” “singing” and “listening.” It is clear that Andy was able to achieve the lowest latency period average during “listening” at 4.7 s; however, the slightly increasing trend line indicates that his response pattern during “singing” was not consistent enough to yield a decreasing trend line.

### Alex

Alex’s latency of responding to target words had a mean of 6 s during baseline, as displayed in Figure 3. His average latency slightly decreased to 5.8 s during the three musical intervention conditions collectively. Visual analysis of his latency responding graph showed a decrease in his response time upon the implementation of only the “singing” intervention (see Figure 2). Alex’s mean scores were 6 s during the “music and singing” and “listening” interventions and 5.6 s during the “singing” intervention. His responding time during the “music and singing” and “listening” interventions did not vary but remained at 6 s throughout. His responding time during the “singing” intervention varied from 6 to 4 s, with 6 s being the most frequent latency of response time. Visual inspection of Alex’s latency for response graph reveals a slightly descending trend for “singing,” but showed neutral trends for “music and singing” nor “listening” demonstrating his unresponsiveness during the latter interventions. His response pattern during “singing” was not consistent enough to yield a clear decreasing trend line.

Regarding overlap, all three participants attained a percentage of non-overlapping data (PND) score below 50% for both “singing and music” and “singing” interventions. On the other hand, the “listening” intervention yielded PND scores of 85% for Andy and 60% for Youssef, suggesting that this form of music produced more positive effects for the two participants compared to the other musical interventions.

### Index of Happiness

**FIGURE 4:**
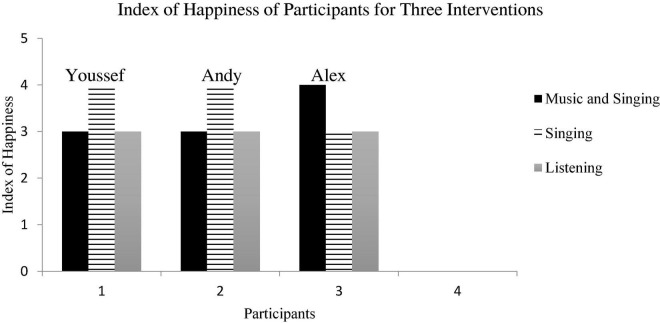
Index of happiness for three music interventions.

Each child showed a different preference while they participated in each of the three musical interventions (see Figure 4). For Youssef and Andy, more smiles and laughter were observed during their sessions with “singing” in comparison to baseline, “listening,” and “music and singing” conditions. As for Alex, he was observed to be more happily engaged during “music and singing” in comparison to the other two intervention conditions and baseline phase. Not one child scored in the unhappiness range (0–2) for this intervention.

### Social Validity

In summary, the intervention period was too short of proving what the long-term outcome concerning verbal behavior would be. However, teachers and parents separately agreed that the procedures used in this intervention were easy and feasible if they were to implement themselves. In terms of outcomes and goals, the majority of respondents rated the intervention as “very acceptable.” Overall, teachers and parents recommended the use of this intervention in the school curriculum since they have separately observed in the home and school setting that music is an activity that excites their children and makes them happier overall.

## Discussion, Conclusion, and Recommendations

### Discussion

Findings from the present study demonstrated that there was a differential effect among the three musical interventions regarding latency in verbal expression for the three participants. Additionally, all three participants were observed as occasionally smiling and even laughing during specific interventions suggesting that these interventions were an overall enjoyable experience.

A finding from this study is that “listening” was the most successful intervention for Andy and Youssef in terms of lowering the latency in verbal expression. As for Alex, “singing” intervention was associated with the lowest mean latency to produce an accurate verbal response. The three participants achieved the lowest mean scores during “music and Singing” as shown in Figure 3. The positive outcomes for Alex are consistent with findings from Quinn (2016). The study by Quinn (2016) explored whether singing could motivate auditory attentiveness in children on the spectrum as compared to regular speech. A finding from this study was that some children with ASD did have increased attention and increased engagement when the information was presented through singing as opposed to speaking (Quinn, 2016). In both studies, “singing” was shown to be a motivating stimulus for children toward achieving a response. Yet, determining on an individualized basis, whether singing or other musical techniques can help toward attaining verbal expression will differ depending on each child’s personal preference (Iovannone et al., 2003).

Another finding from this study is that children may respond differently to various musical strategies based on their inherent skills (Figure 2). In this study, the lowest overall mean scores and decreasing trend lines were associated with the implementation of the “listening” intervention for both Andy and Youssef suggesting that both benefited the most from this intervention. This could be attributed to the fact that Youssef and Andy started with higher verbal abilities than Alex. Overall, Youssef excelled during “singing” and “listening” interventions beyond the other two participants. Although he began the intervention at the same time as the two other children, he finished first learning all the words to the songs and completing the intervention. Youssef’s mother had commented in the survey about how he uses his incredible music memory to fill in words to songs that he had heard only once or twice, perhaps having to do with the musical pitch of the words. This finding agrees with Stanutz et al. (2014), who demonstrated that children with ASD particularly excelled in better pitch discrimination abilities and had better long-term memories for melody without any previous musical training compared to peers of the same age.

One more finding from this study is that all three children were found to be either neutral or happy during the music intervention. Because children with ASD were found to enjoy leisure activities that require less demanding social interaction (Eversole et al., 2016), perhaps participation in this music activity was not particularly demanding.

An implication that can be drawn from this study is that not only do children with ASD show limited processing of social and more complex sounds like connected speech but also they neurologically do not perceive human speech as socially rewarding as typically developing children (Chevallier et al., 2012). Due to this fact, children with ASD are not as attentive or responsive to human speech (Abrams et al., 2013). This signifies that speech by itself is not interesting enough for children with ASD. Another study by Kuhl et al. (2005) found that children with ASD demonstrated a preference for non-speech sounds in contrast to children not on the spectrum. Like the teaching *via* this intervention, melodically delivered speech can be generalized to more settings when non-verbal children with ASD were taught words to elicit (Sandiford et al., 2013).

### Conclusion

While all children have their preferences, learning about activities that bring enjoyment to children with ASD or speech difficulties can provide better intervention planning (Eversole et al., 2016). Using this music approach as a technique to encourage verbal expression supported the fact that using an intervention that teaches responding with an immediate and high reinforcement even at attempts at speech may demonstrate the connection between responding and reinforcement (Koegel et al., 2001). “Singing” was seen to be both motivational and reinforcing enough to produce responses from the children lowering the overall latency periods preceding verbal expression. Because communication is a troublesome area for children on the spectrum, there is a need for creative and enjoyable interventions in this field. The flexibility of this intervention allows it to be used in many environments with many different people, among those parents, therapists, music teachers, and special education teachers.

Results from the current study found that “listening” proved to be the most successful in terms of producing the quickest responses for two participants. Although the effect of the “singing” intervention varied among participants with some achieving lower mean scores than others, the promising effect of the intervention exists. The happiness levels of the students ranged from neutral to happy, signifying an overall positive experience while they were participating in the music ABA intervention. Using activities preferred by children is valuable in motivating children, especially in areas of difficulty (Eversole et al., 2016).

Findings from the intervention show that teaching verbal expression through a musical strategy is an overall positive experience for children on the autism spectrum. Children may have different strengths, including musical pitch-discrimination, which may enhance their performance through musical interventions. However, the musical preference of each child must always be considered.

### Limitations of the Study

The current study investigates an understudied area of intervention for children with ASD; however, there are some limitations to be considered. First, the small number of participants constrains our findings, however, this is the case with single-subject design studies. Thus, results from such studies are not claimed to be generalizable to all situations or individuals. Second, the researchers did not have the luxury to select the study participants from a large pool of potential participants, since Lebanon has a very small number of centers specialized for children with autism. Lastly, the three participants exhibited a varied degree of challenging behaviors, which required the interruption of a few sessions. The interventionist was able to redirect the participants’ behaviors back to the session; however, the occurrence of the challenging behaviors may have disrupted the flow of the intervention.

### Recommendations for Future Studies

Because this study was able to show that using a structured combination of ABA and music with verbal and non-verbal children on the spectrum could teach verbal expression, further research should be conducted in this area. Considering that, “listening” produced the best responses compared to “singing and music” and “singing,” further research conducted using music with children on the spectrum should focus on the engagement of children with different musical approaches and verbal expression. Perhaps using a single-subject design such as a multiple-baseline design for “listening” for multiple participants would reassure that the effects are due to the intervention, not the children’s preferences.

As mentioned in the limitations section, the challenging behaviors exhibited by the three participants disrupted the flow of the intervention on multiple occasions. Researchers are encouraged to study challenging behavior as an additional dependent variable when implementing a music intervention. Lower levels of challenging behaviors during a music intervention would be valuable data to demonstrate that the intervention has a positive effect on both verbal expression and task enjoyment.

Another suggestion for future research is testing whether this approach works as well for children with other types of disabilities (e.g., children with ADHD, intellectual disabilities, speech, and language impairments). While this study focused on a younger age group (6 years old), future research examining the effects of such intervention with other age groups is warranted.

Researchers attempting to replicate the present study are encouraged to have two implementers conducting the intervention sessions. The sessions should include an individual actively playing the music and another individual situated beside the child to alleviate multitasking on one individual. This will keep the sessions running smoothly and keep the child on task throughout the entire intervention session.

### Recommendations for Practice

Results from this study may help special education teachers, ABA therapists, or parents of children with ASD implement effective techniques for targeting verbal expression in their children with adequate knowledge about the emotional experience the particular intervention can bring. Currently, ABA-based interventions are widely conducted to teach individuals with ASD verbal and non-verbal expression. Based on findings from the present study, practitioners should consider combining ABA interventions with music therapy components to increase treatment effectiveness. Additionally, the aspect of measuring participants’ affective behavior (i.e., index of happiness) in this study may help practitioners uncover which type of music intervention is most enjoyable for each child. Exploring measures such as indices of happiness may be helpful for practitioners as they select effective interventions for individuals with ASD, as many of them may have difficulties communicating in words whether or not they find the implemented interventions enjoyable.

## Data Availability Statement

The raw data supporting the conclusions of this article will be made available by the authors, without undue reservation.

## Ethics Statement

The studies involving human participants were reviewed and approved by the American University of Beirut, IRB. Written informed consent to participate in this study was provided by the participants’ legal guardian/next of kin. Written informed consent was obtained from the individual(s), and minor(s)’ legal guardian/next of kin, for the publication of any potentially identifiable images or data included in this article.

## Author Contributions

All authors listed have made a substantial, direct, and intellectual contribution to the work, and approved it for publication.

## Conflict of Interest

The authors declare that the research was conducted in the absence of any commercial or financial relationships that could be construed as a potential conflict of interest.

## Publisher’s Note

All claims expressed in this article are solely those of the authors and do not necessarily represent those of their affiliated organizations, or those of the publisher, the editors and the reviewers. Any product that may be evaluated in this article, or claim that may be made by its manufacturer, is not guaranteed or endorsed by the publisher.
