# Supplementary material for: Effects of Three Music Therapy Interventions on the Verbal Expressions of Children With Autism Spectrum Disorder: A Combined Single-Subject Design
Source: Front Psychol. 2022 Mar 4;13:819473. doi: 10.3389/fpsyg.2022.819473 (PMC8931400; doi:10.3389/fpsyg.2022.819473)
Supplement: Supplementary file 1 [file Data_Sheet_1.pdf]

Social Validity Questionnaires

Teacher Questionnaire

Please rate this music-ABA intervention technique from 1-8 with, 1 corresponding to “not at all acceptable” and 8 being “very acceptable”. Thank you ☺

1. How acceptable do you find this treatment to be for the student’s verbal behavior?

   ______ ______ ______ ______ ______ ______ ______

   Not at all acceptable | moderately acceptable | very acceptable

2. How willing would you be to carry out this procedure yourself if you had to change the student’s verbal behavior?

   ______ ______ ______ ______ ______ ______ ______

   Not at all acceptable | moderately acceptable | very acceptable

3. How much do you like the procedures used in this treatment?

   ______ ______ ______ ______ ______ ______ ______

   Not at all acceptable | moderately acceptable | very acceptable

4. How likely is this treatment to make permanent improvements in the student?

   ______ ______ ______ ______ ______ ______ ______

   Not at all acceptable | moderately acceptable | very acceptable

5. To what extent or undesirable side effects likely to result from this treatment?

   ______ ______ ______ ______ ______ ______ ______

   Not at all acceptable | moderately acceptable | very acceptable

6. Overall, what is your general reaction to this form of treatment?

_______ _______ _______ _______ _______ _______ _______

Not at all acceptable | moderately acceptable | very acceptable

7. I would suggest the use of this intervention to other special education or music teachers

_______ _______ _______ _______ _______ _______ _______

Not at all acceptable | moderately acceptable | very acceptable

8. Most special education or music teachers would find this intervention suitable for problem behaviors.

_______ _______ _______ _______ _______ _______ _______

Not at all acceptable | moderately acceptable | very acceptable

9. I would be willing to use this intervention in the special education setting

_______ _______ _______ _______ _______ _______ _______

Not at all acceptable | moderately acceptable | very acceptable

10. This intervention would be appropriate for a variety of children

_______ _______ _______ _______ _______ _______ _______

Not at all acceptable | moderately acceptable | very acceptable

11. Overall, this intervention was beneficial for the child

_______ _______ _______ _______ _______ _______ _______

Not at all acceptable | moderately acceptable | very acceptable

12. How important do you think it is that you child's school incorporate this intervention into their curriculum?

_______ _______ _______ _______ _______ _______ _______

Not at all | moderately | very

acceptable	acceptable	acceptable

Thank you ☺

Appendix B

A. Parent Questionnaire

How old is your child? _______________

What grade is your child in? _______________

What is your child's IQ range?______________

Does the child have any speech problems?

____

Does your child benefit from speech therapy?

Does the child have any visual problems? ________________________

If so how severe are they? ____

Does the child have hearing problems? _____ If so, how severe are his hearing problems?

____

Is the child receiving any special school services such as special education, reading services, tutoring etc.? If yes, can you please mention them? _______________________

____

____

What is the main language spoken at home? _____________________

If it is a language other than English, describe the extent to which it is spoken ____

What is the child's primary language? ________________________________

Does your child have educational difficulties? _______If so, in what? ____

Has your child had previous experience in music therapy? ______
